# Endoscopic ultrasound-guided left gastric vein embolization for esophageal and gastric varices in liver cirrhosis: A case report

**DOI:** 10.1097/MD.0000000000050710

**Published:** 2026-09-11

**Authors:** Hongxue Lu, XinYao Yang, Chao Huang, Di Zhang, Pu Wang

**Affiliations:** a Department of Gastroenterology, Sichuan Provincial People’s Hospital, School of Medicine, University of Electronic Science and Technology of China, Chengdu, Sichuan, China.

**Keywords:** embolization, endoscopic ultrasound, gastroesophageal varices, left gastric vein, liver cirrhosis

## Abstract

**Rationale:**

Conventional endoscopic ultrasound (EUS)-guided therapy for upper gastrointestinal varices mainly fills the variceal lumen without interrupting the feeding vessels, and recurrence remains common. We report a case in which EUS-guided embolization of the left gastric vein, the varices feeding vessel, was performed for esophageal and gastric varices.

**Patient concerns:**

A 47-year-old man with decompensated cirrhosis presented with hematemesis and melena.

**Diagnosis:**

Contrast-enhanced abdominal computed tomography showed decompensated cirrhosis with portal hypertension. Upper gastrointestinal endoscopy revealed esophageal varices extending below the cardia, 1.5 to 2 cm in diameter and consistent with gastroesophageal varices type 1, with red color signs. Laboratory findings included hemoglobin 57 g/L, albumin 28.8 g/L, and prothrombin time 18.7 seconds.

**Interventions:**

Endoscopy showed gastroesophageal varices type 1. EUS identified multiple perforating veins, all originating from the left gastric vein (LGV). The LGV was punctured with a 22-gauge needle, followed by deployment of an 8-mm coil and injection of 30 mL Lauromacrogol into the adjacent para-esophageal vein.

**Outcomes:**

Blood flow in the LGV was abolished, and the flow velocity within the esophageal varices decreased from 7 to 0–1 cm/s. No rebleeding or adverse events occurred during 4 months of follow-up, and endoscopy confirmed marked attenuation of the varices.

**Lessons:**

EUS-guided coil embolization of the LGV is feasible, safe, and effective for esophageal and gastric varices in cirrhosis and warrants evaluation in larger studies.

## 1. Introduction

Endoscopic ultrasound (EUS)-guided treatment of upper gastrointestinal varices has demonstrated superior efficacy compared with conventional endoscopic therapy.^[1,2]^ However, current EUS-guided techniques still rely on filling the variceal lumen and do not fundamentally interrupt the blood supply of the varices, resulting in recurrence rates of 14% to 30%.^[3]^ EUS can precisely map the portal venous system and the perforating vessels that feed the varices, allowing targeted occlusion of these feeding vessels, which has been reported to achieve favorable outcomes.^[4,5]^ Here, we present a case of successful EUS-guided occlusion of the left gastric vein (LGV) for the treatment of gastroesophageal varices type 1.

## 2. Case presentation

**Figure 1.**
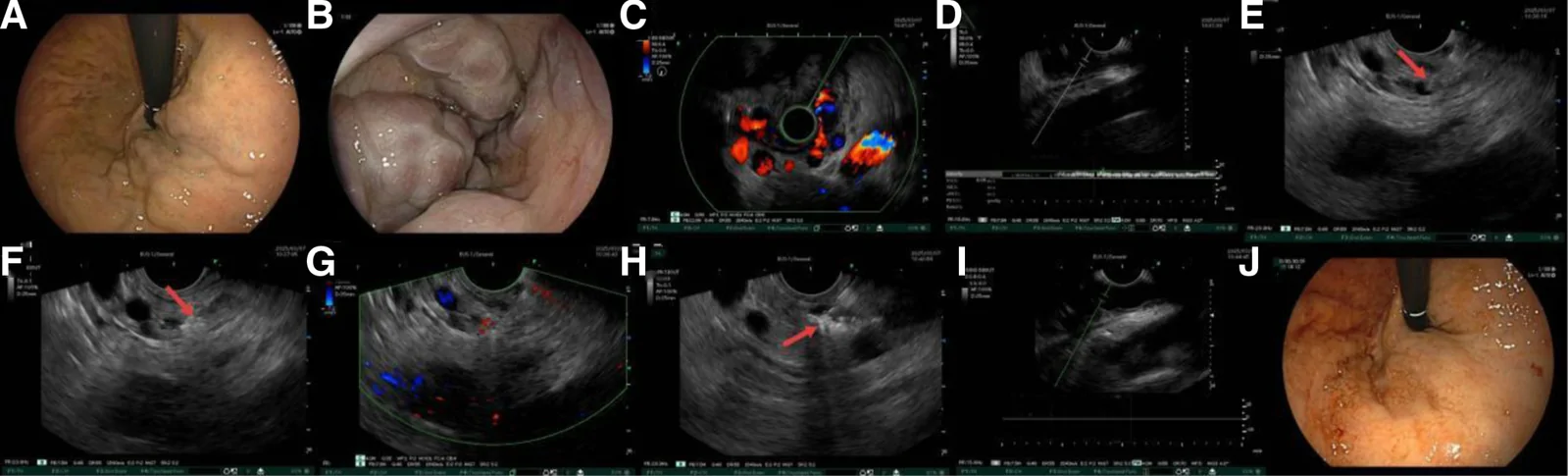
(A and B) Esophageal and gastric varices on white-light endoscopy. (C) Perforating vein on radial EUS. (D) Preoperative measurement of flow velocity (7 cm/s) in the esophageal varices. (E) A 22-gauge needle puncturing the left gastric vein (arrow). (F) Deployment of the coil (arrow). (G) Doppler imaging confirming the absence of blood flow signals around the coil. (H) Injection of the lauromacrogol. (I) Flow velocity of the esophageal varices decreased to 0 to 1 cm/s. (J) White-light imaging 10 minutes after the procedure showing attenuation of the gastric varices.

A 47-year-old man was admitted with hematemesis and melena. Contrast-enhanced abdominal computed tomography showed decompensated cirrhosis with portal hypertension. Upper gastrointestinal endoscopy revealed esophageal varices (EV) extending below the cardia, 1.5 to 2 cm in diameter and consistent with gastroesophageal varices type 1, with red color signs (Fig. 1A and B). Laboratory findings included hemoglobin 57 g/L, albumin 28.8 g/L, and prothrombin time 18.7 seconds.

Radial EUS revealed 2 perforating veins, 2 to 3 mm in diameter, below the level of the cardia and a 2-mm perforating vein in the lower esophagus (Fig. 1C). Color Doppler confirmed an afferent (inflow) direction in all of these veins, with a predominant red signal when the perforating veins were oriented perpendicular to the EUS probe. Linear EUS showed that all the perforating veins originated from the left gastric vein, which exhibited the same inflow signal on Doppler imaging. Before treatment, the blood flow velocity was 6 to 8 cm/s in the LGV (Supplementary Material 2, Supplemental Digital Content 1) and 7 cm/s within the EV. The diameter of LGV was 4.2 mm (Fig. 1D).

Due to the multiple perforating veins shared a common origin, we selected to occlude the LGV trunk. At the level of the cardia, the LGV was punctured with a 22-gauge needle (Fig. 1E), followed by deployment of one 8-mm coil (Cook Medical) (Fig. 1F). Ten minutes later, the Doppler signal and flow velocity in the LGV (Fig. 1G) and the para-esophageal veins were barely detectable (Supplementary Materials 2 and 3, Supplemental Digital Contents 2). After the blood flow had slowed, 30 mL of lauromacrogol was injected into the para-esophageal vein closest to the varices under EUS guidance (Fig. 1H and Supplementary Material 1, Supplemental Digital Content 3). The blood flow velocity within the EV then decreased to 0 to 1 cm/s (Fig. 1I). White-light imaging 10 minutes after the procedure showed attenuation of the gastric varices (GV) (Fig. 1J).

No rebleeding or other adverse events occurred during the procedure or throughout the 4-month follow-up. White-light endoscopy showed marked regression of the GV and a slight reduction in the size of the EV. EUS showed acoustic shadowing of the coil within the LGV, with no detectable Doppler signal, confirming complete occlusion of the vessel. No significant blood flow signals were detected within the para-esophageal veins. The blood flow velocity of the EV remained 0 to 1 cm/s, and the soft texture of the varices indicated a marked reduction in pressure. An additional 20 mL of lauromacrogol was injected under direct endoscopic visualization, and no significant bleeding occurred during needle withdrawal (Supplementary Material 4, Supplemental Digital Content 4).

## 3. Discussion

EUS-guided occlusion of the feeding vessels has emerged as a research focus in the management of gastrointestinal varices, and reported cases have shown favorable outcomes for GV.^[4,5]^ In contrast, complete elimination of EV by major feeding-vessel occlusion solely has rarely been reported, and in our experience its effect is less satisfactory than that for GV. This discrepancy is likely attributable to the multiple feeding sources of EV. Therefore, although occlusion of the LGV, the dominant feeder, is not always sufficient to eliminate EV completely, the resulting reduction in intravariceal pressure and flow velocity remains clinically important as it plays a cornerstone role for safer and more effective subsequent sclerotherapy.

In conclusion, EUS-guided LGV embolization for esophageal and GV in cirrhosis has the potential to become a cost-effective, safe, and effective treatment. Standardized protocols for this technique should be established, and larger-scaled studies are warranted.

## Acknowledgments

The authors thank Dr Tao Lu (Department of Radiology, Sichuan Provincial People’s Hospital) for insightful perspectives on the hemodynamics of portal hypertension; Dr Lu has given permission to be named. This work was supported by the Sichuan Science and Technology Program (2021YFS0375). The authors declare no conflicts of interest.

## Author contributions

**Conceptualization:** Pu Wang.

**Data curation:** Pu Wang.

**Writing – original draft:** Hongxue Lu.

**Writing – review & editing:** XinYao Yang, Chao Huang, Di Zhang, Pu Wang.








