# Supplementary material for: Endoscopic ultrasound-guided left gastric vein embolization for esophageal and gastric varices in liver cirrhosis: A case report
Source: Medicine (Baltimore). 2026 Sep 11;105(37):e50710. doi: 10.1097/MD.0000000000050710 (PMC13574429; doi:10.1097/MD.0000000000050710)
Supplement: Supplementary file 1 [file medi-105-e50710-s001.pdf]

Supplementary material 2.1

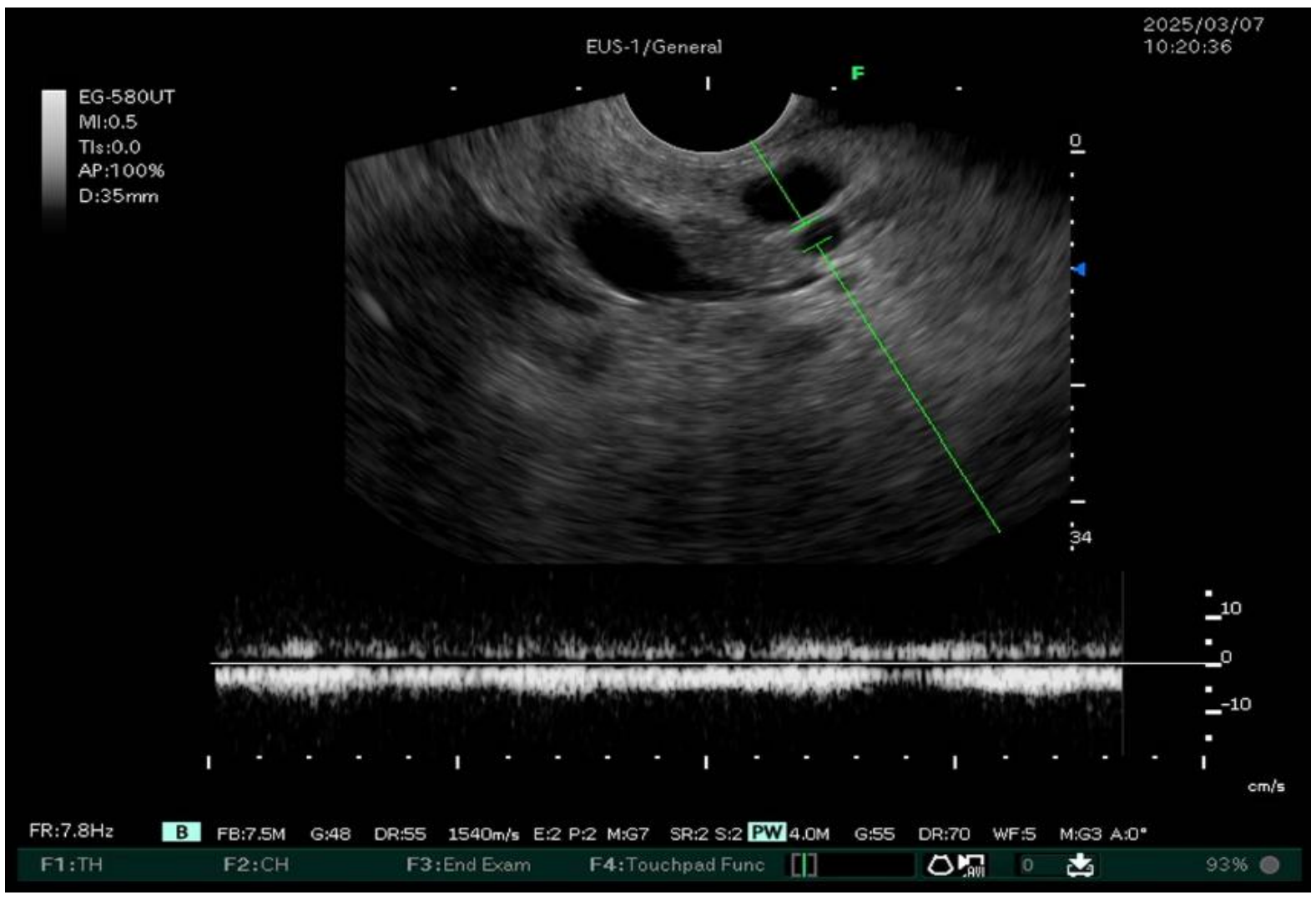

Preoperative measurement of flow velocity (6-8cm/s) in the left gastric vein.

Supplementary material 2.2

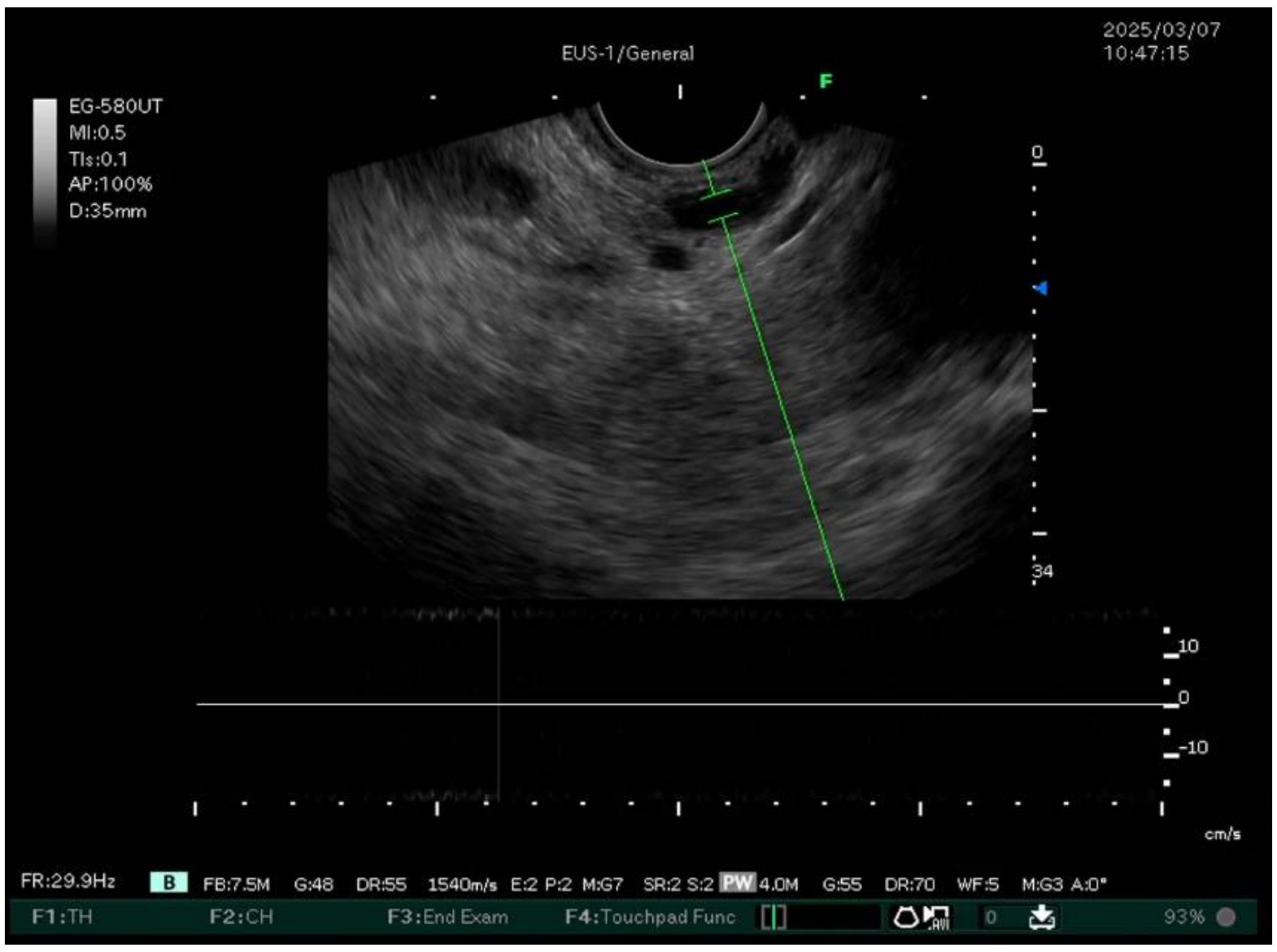

The blood flow velocity of the left gastric vein was 0 cm/s.
