# Supplementary material for: Endoscopic ultrasound-guided left gastric vein embolization for esophageal and gastric varices in liver cirrhosis: A case report
Source: Medicine (Baltimore). 2026 Sep 11;105(37):e50710. doi: 10.1097/MD.0000000000050710 (PMC13574429; doi:10.1097/MD.0000000000050710)
Supplement: Supplementary file 2 [file medi-105-e50710-s002.pdf]

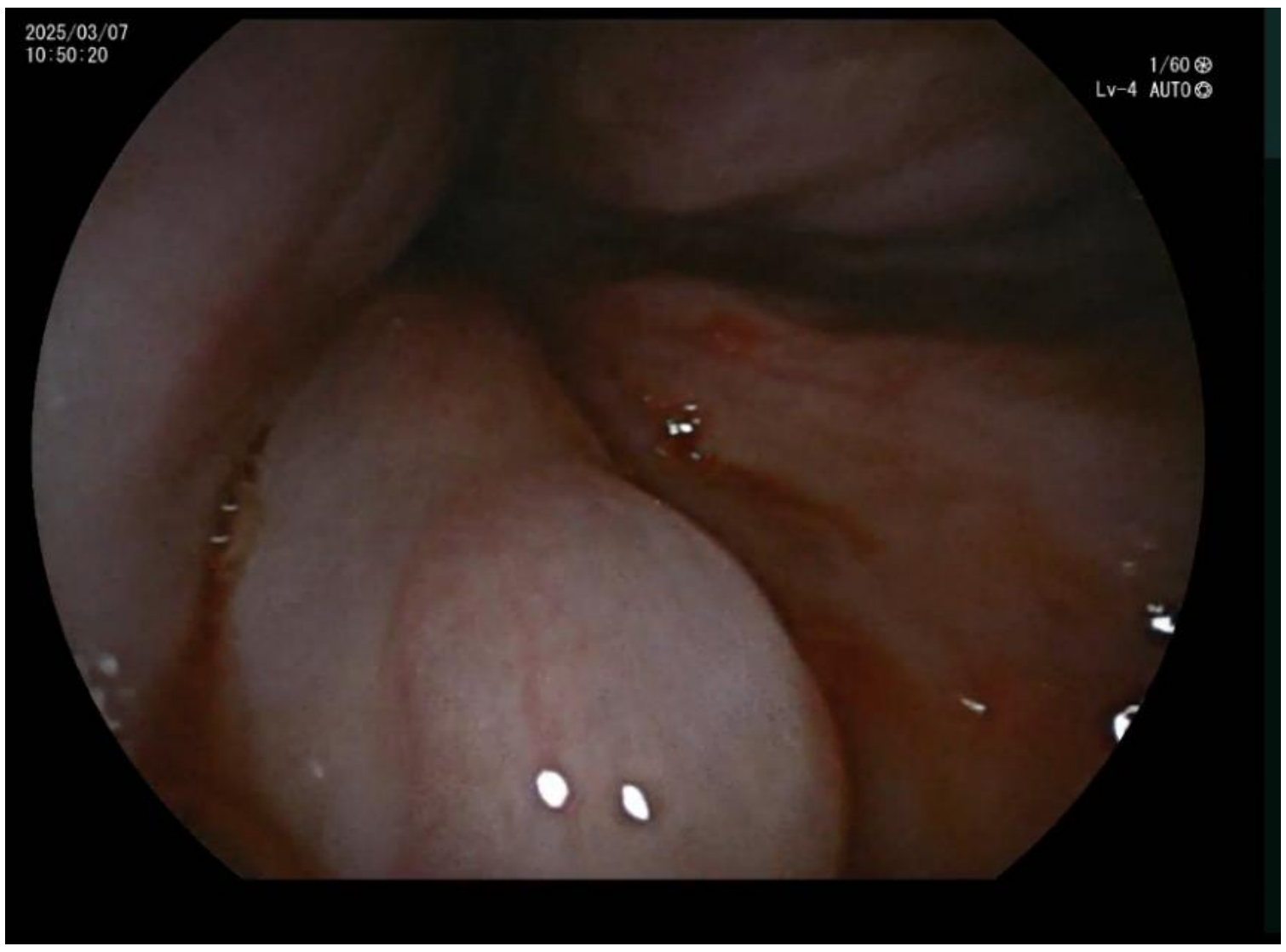
2025/03/07
10:50:20
1/60
Lv-4 AUTO
