# Supplementary material for: Endoscopic ultrasound-guided left gastric vein embolization for esophageal and gastric varices in liver cirrhosis: A case report
Source: Medicine (Baltimore). 2026 Sep 11;105(37):e50710. doi: 10.1097/MD.0000000000050710 (PMC13574429; doi:10.1097/MD.0000000000050710)
Supplement: Supplementary file 3 [file medi-105-e50710-s003.pdf]

Supplementary material 4.1

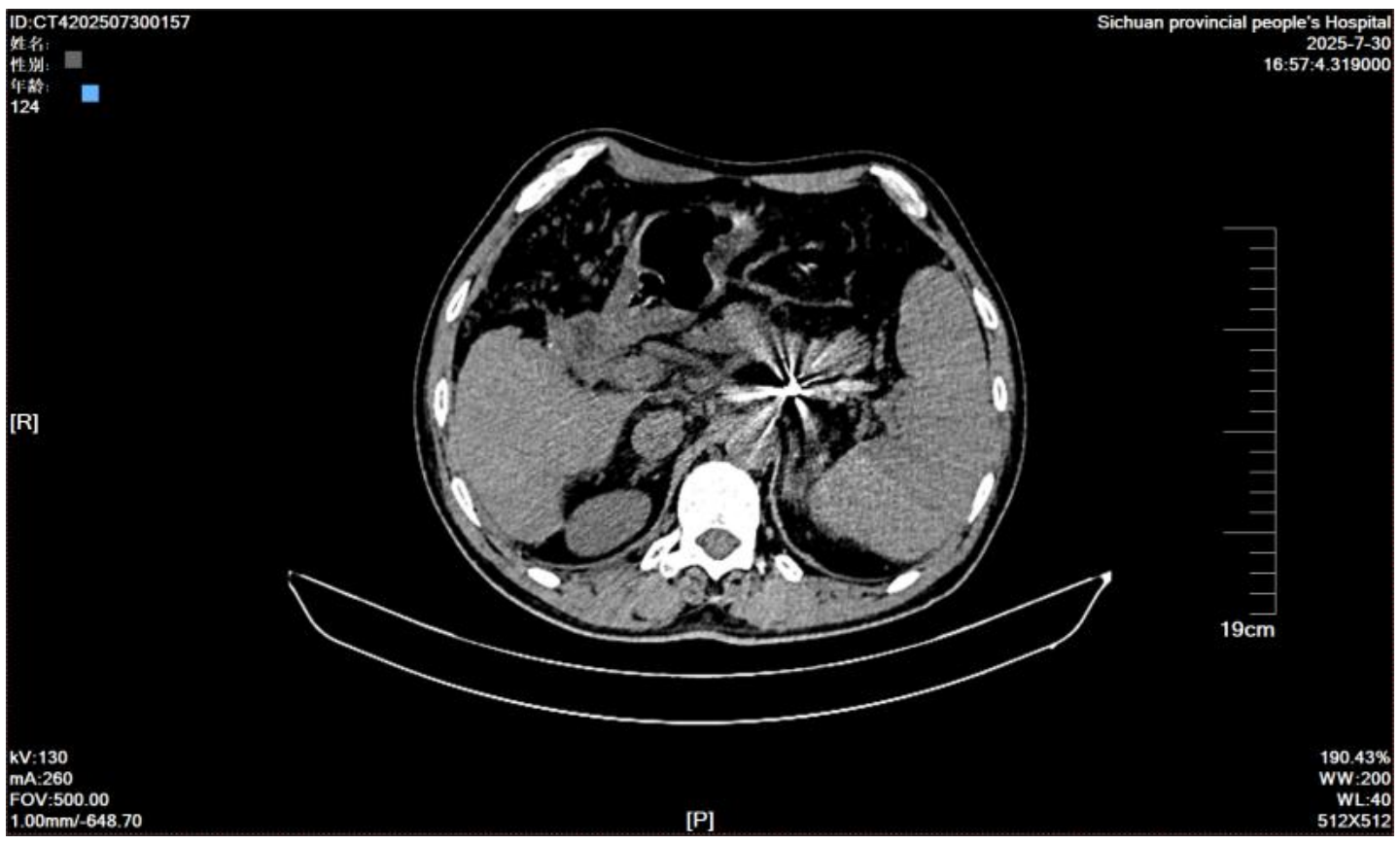

CT revealed no displacement of the coil.

Supplementary material 4.2

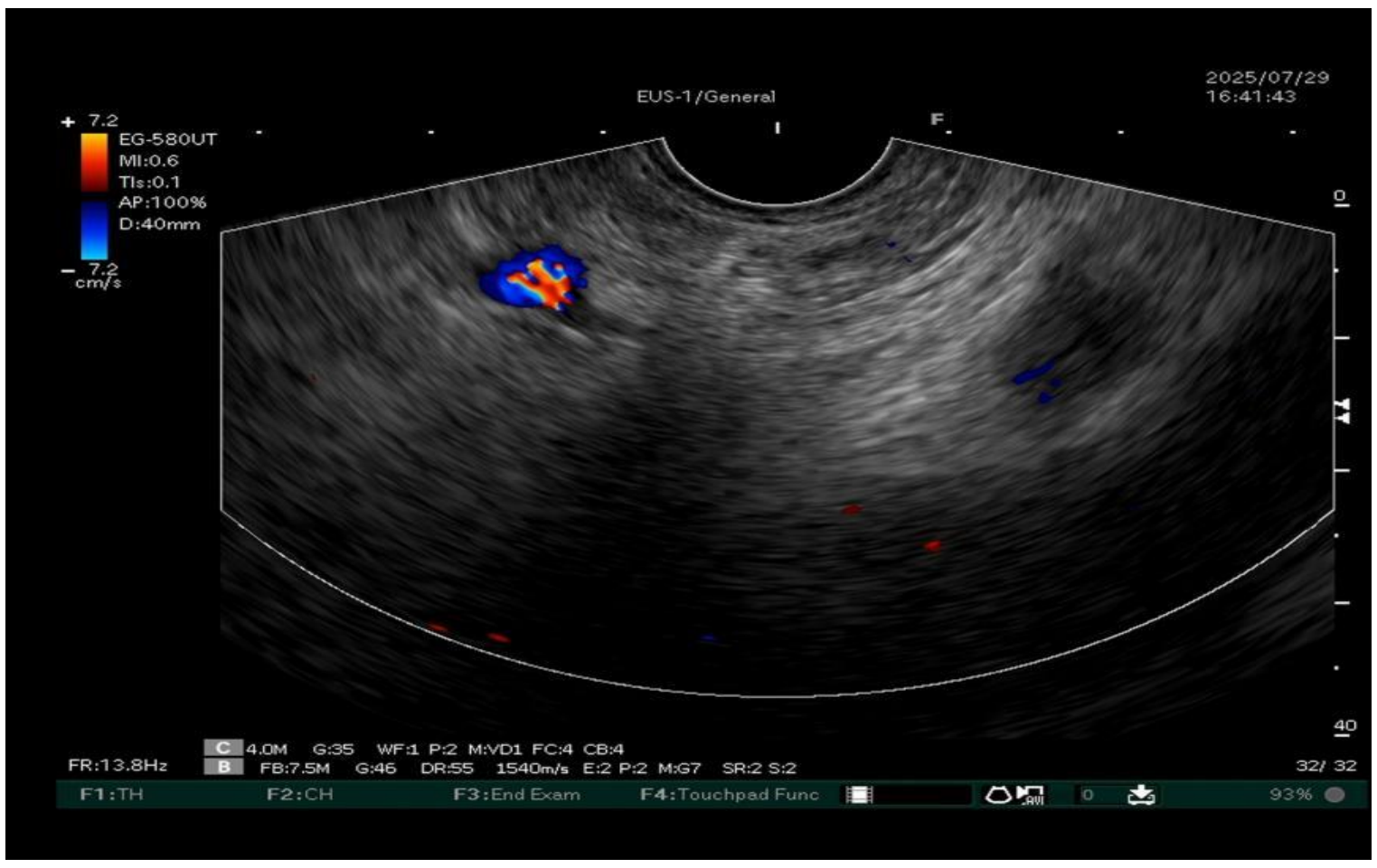

EUS revealed no displacement of the coil.

Supplementary material 4.3

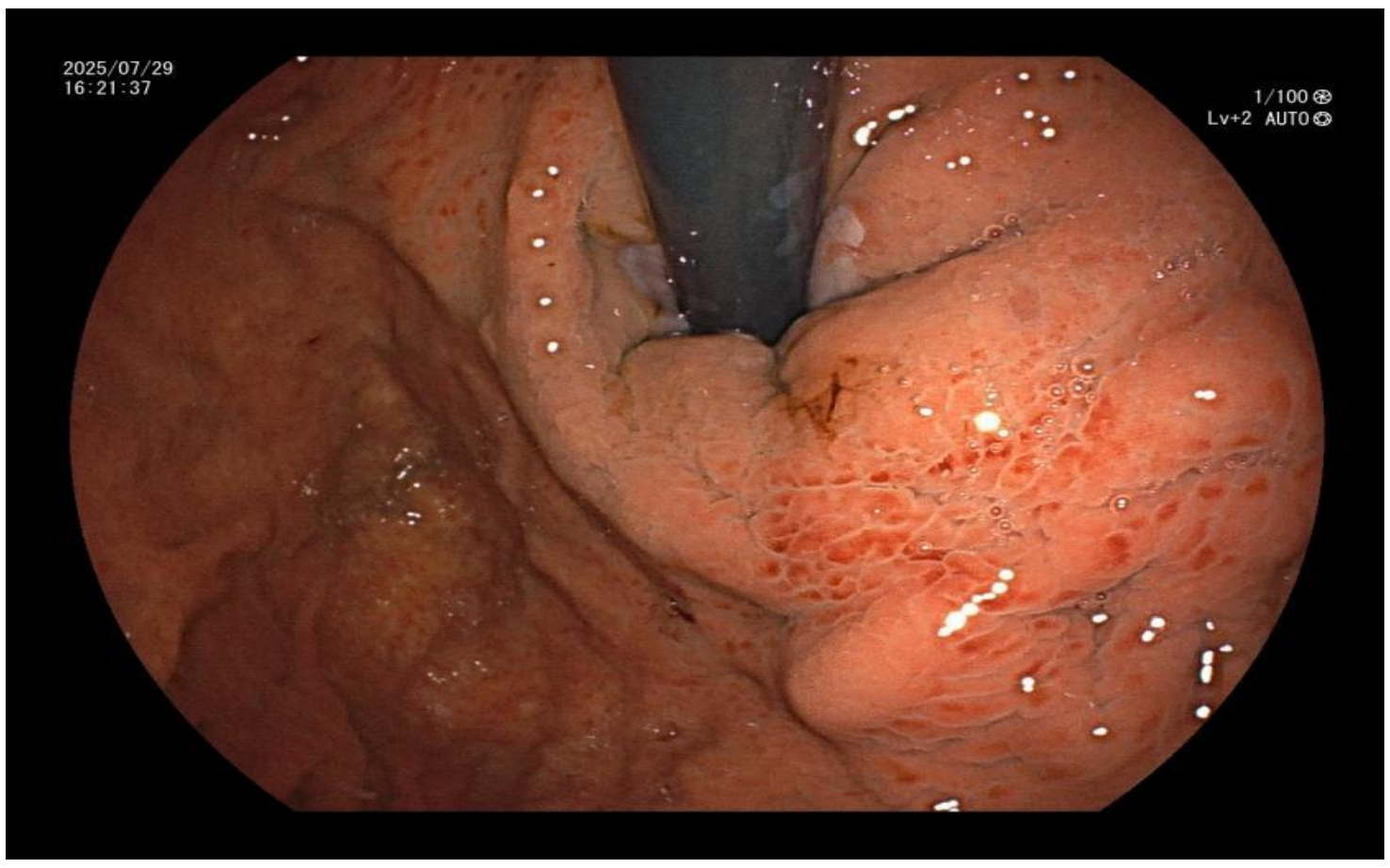

Gastric varices 4 months later disappearance.

Supplementary material 4.4

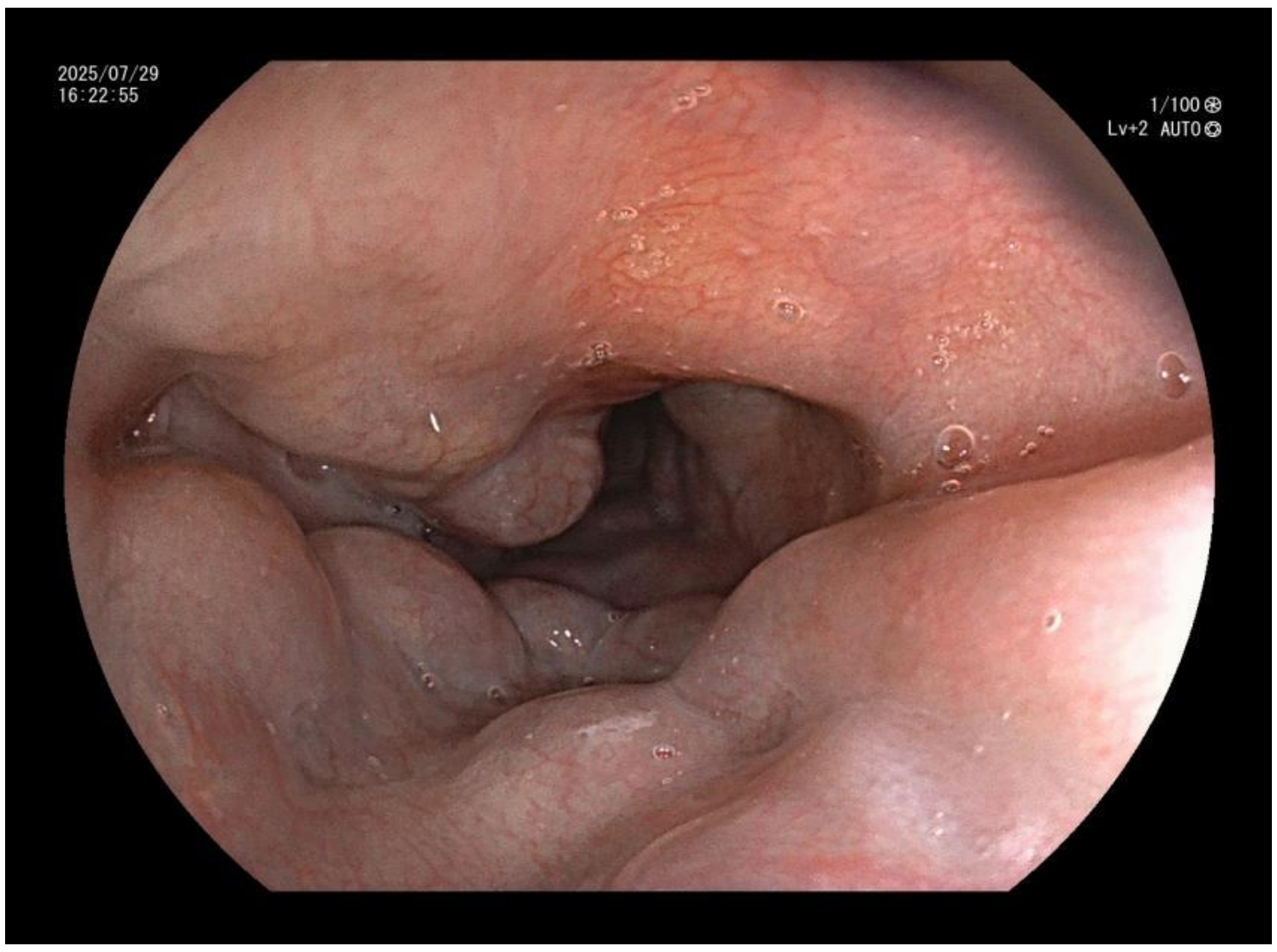

Esophageal varices 4 months later smaller in size and softer in touch.

Supplementary material 4.5

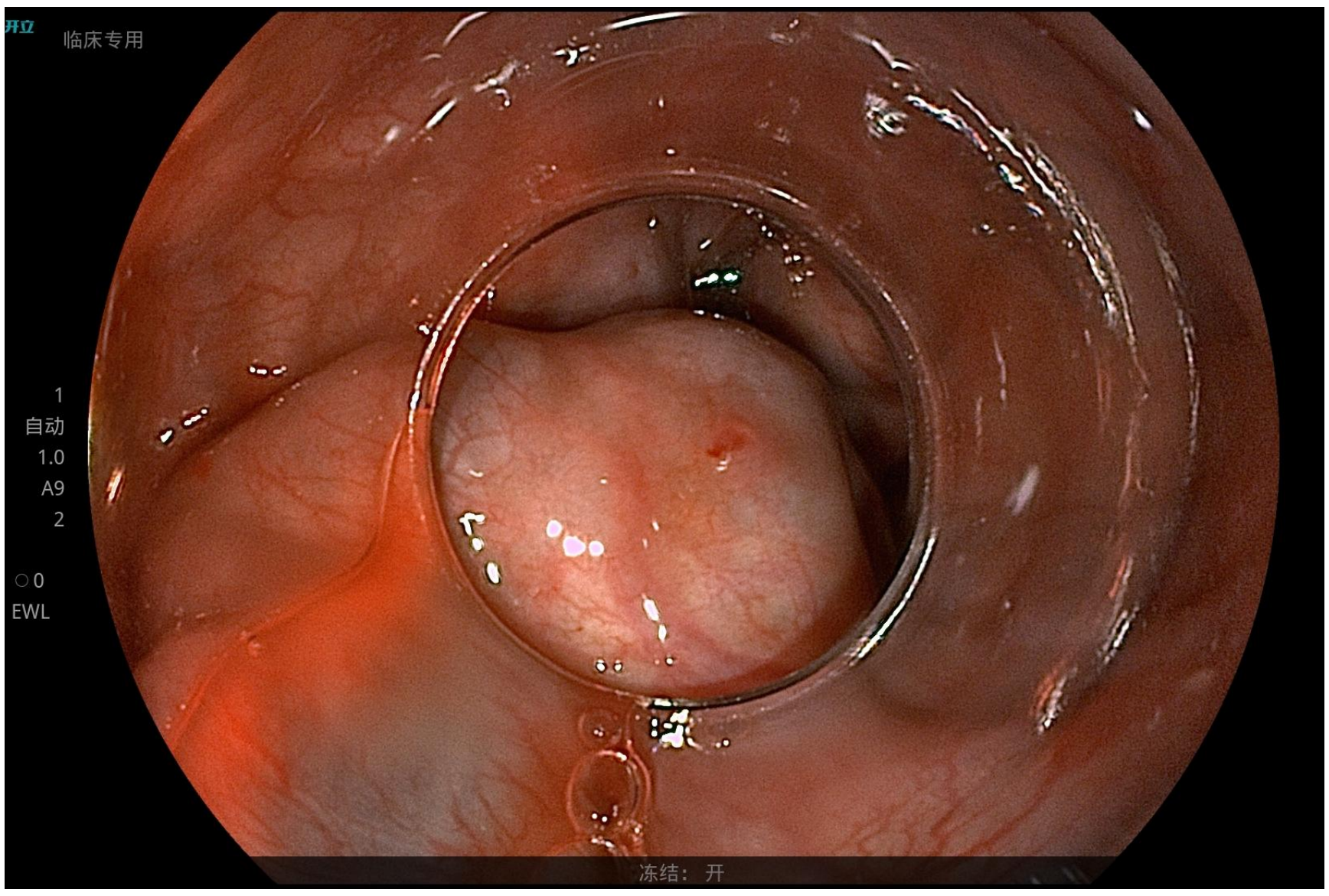

4 monthslater, after supplementary injection of sclerosant , no significant bleeding occurred during needle withdrawal after injection completion.
