# Supplementary material for: Endoscopic ultrasound-guided left gastric vein embolization for esophageal and gastric varices in liver cirrhosis: A case report
Source: Medicine (Baltimore). 2026 Sep 11;105(37):e50710. doi: 10.1097/MD.0000000000050710 (PMC13574429; doi:10.1097/MD.0000000000050710)
Supplement: Supplementary file 4 [file medi-105-e50710-s004.pdf]

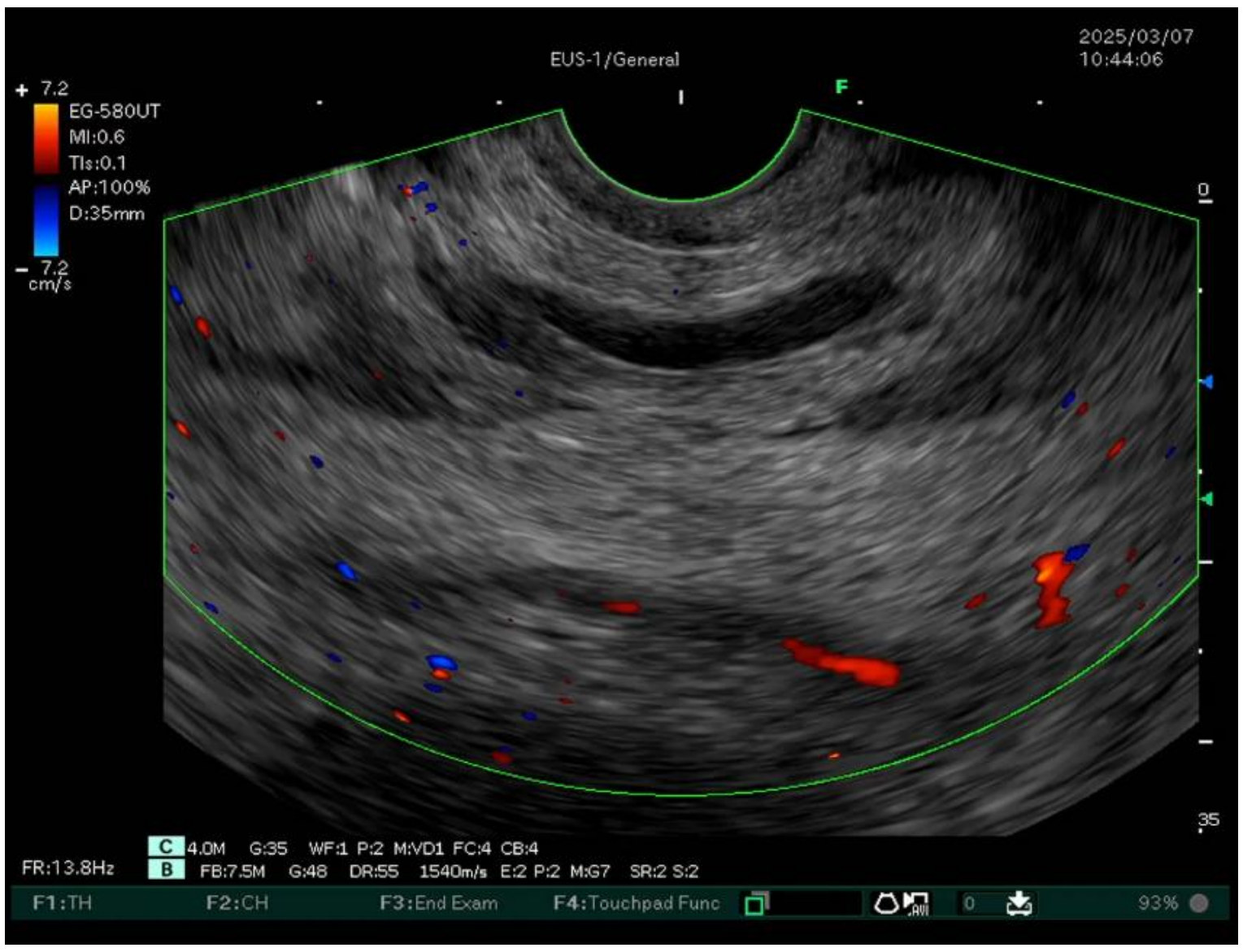
EUS-1/General
2025/03/07
10:44:06
+ 7.2
EG-580UT
MI:0.6
TIs:0.1
AP:100%
D:35mm
− 7.2
cm/s
F
0
35
FR:13.8Hz
C 4.0M G:35 WF:1 P:2 M:VD1 FC:4 CB:4
B FB:7.5M G:48 DR:55 1540m/s E:2 P:2 M:G7 SR:2 S:2
F1:TH
F2:CH
F3:End Exam
F4:Touchpad Func
0
93%
